# Progress Toward Poliomyelitis Eradication — Nigeria, January 2018–May 2019

**DOI:** 10.15585/mmwr.mm6829a3

**Published:** 2019-07-26

**Authors:** Usman S. Adamu, W. Roodly Archer, Fiona Braka, Eunice Damisa, Anisur Siddique, Shazad Baig, Jeffrey Higgins, Gerald Etapelong Sume, Richard Banda, Charles Kipkoech Korir, Ndadilnasiya Waziri, Saheed Gidado, Philip Bammeke, Aboyowa Edukugo, Gatei wa Nganda, Joseph C. Forbi, Cara C. Burns, Hongmei Liu, Jaume Jorba, Adeyelu Asekun, Richard Franka, Steven G.F. Wassilak, Omotayo Bolu

**Affiliations:** ^1^Polio Emergency Operations Center, National Primary Health Care Development Agency, Abuja, Nigeria; ^2^Global Immunization Division, Center for Global Health, CDC; ^3^Expanded Program on Immunization, World Health Organization Nigeria Country Office, Abuja, Nigeria; ^4^United Nations Children’s Fund Nigeria Country Office, Abuja, Nigeria; ^5^Bill and Melinda Gates Foundation, Abuja, Nigeria; ^6^Division of Emergency Operations, Center for Preparedness and Response, CDC; ^7^National Stop Transmission of Polio Program, Africa Field Epidemiology Network, Nigeria Office, Abuja, Nigeria; ^8^Division of Viral Diseases, National Center for Immunization and Respiratory Diseases, CDC; ^9^CDC Nigeria Country Office, Abuja, Nigeria.

The number of wild poliovirus (WPV) cases in Nigeria decreased from 1,122 in 2006 to six WPV type 1 (WPV1) in 2014 (*1*). During August 2014–July 2016, no WPV cases were detected; during August–September 2016, four cases were reported in Borno State. An insurgency in northeastern Nigeria had resulted in 468,800 children aged <5 years deprived of health services in Borno by 2016. Military activities in mid-2016 freed isolated families to travel to camps, where the four WPV1 cases were detected. Oral poliovirus vaccine (OPV) campaigns were intensified during August 2016–December 2017; since October 2016, no WPV has been detected (*2*). Vaccination activities in insurgent-held areas are conducted by security forces; however, 60,000 unvaccinated children remain in unreached settlements. Since 2018, circulating vaccine-derived poliovirus type 2 (cVDPV2) has emerged and spread from Nigeria to Niger and Cameroon; outbreak responses to date have not interrupted transmission. This report describes progress in Nigeria polio eradication activities during January 2018–May 2019 and updates the previous report (*2*). Interruption of cVDPV2 transmission in Nigeria will need increased efforts to improve campaign quality and include insurgent-held areas. Progress in surveillance and immunization activities will continue to be reviewed, potentially allowing certification of interruption of WPV transmission in Africa in 2020.

## Security Situation

A violent insurgency that arose in 2009 and was followed by insurgents seizing territory beginning in 2012 in Borno, Adamawa, and Yobe states (and bordering areas of Cameroon, Chad, and Niger) led to the internal displacement of 1.8 million persons (*3*). By 2016, this conflict created a humanitarian crisis in which an estimated 468,800 children aged <5 years resided in insurgent-held areas in Borno with no health services, including vaccination and surveillance activities (*2*). Since December 2016, movement of populations within and out of Borno insurgent-held areas has continued with increasing numbers of persons now living in areas outside insurgents’ control; however, many settlements remain inaccessible (Figure).

**FIGURE:**
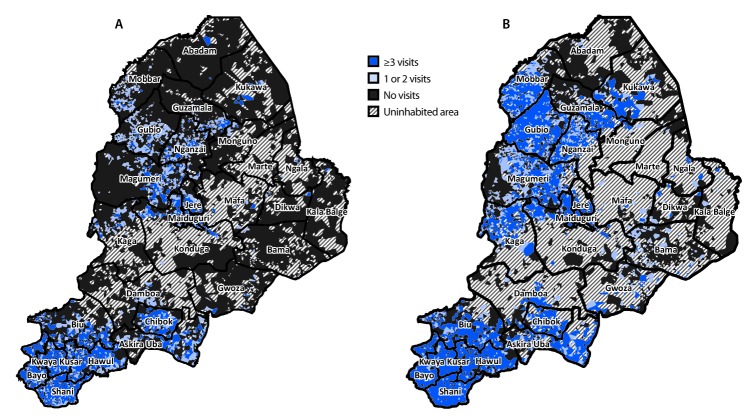
Inhabited settlements reached with bivalent oral poliovirus vaccine using standard house-to-house, Reaching Every Settlement,* and Reaching Inaccessible Children^†^ approaches during August–December 2016 (A) and August 2016–May 2019 (B), by number of cumulative vaccination visits reaching children aged <5 years — Borno State, Nigeria, August 2016–May 2019^§,¶^ * Reaching Every Settlement is an approach in which security escorts enable vaccinators to reach children in insurgent-held areas. ^†^ Reaching Inaccessible Children is an approach in which trained military personnel vaccinate children in settlements that only they can access. ^§^ During August–September 2016, 52.4% of the population resided in settlements reached by vaccination teams three or more times, 15.5% in settlements reached one to two times, and 32.1% in settlements that were not reached; during August 2016–May 2019, 88.3% of the population resided in settlements that were reached three or more times, 4.4% in settlements reached one to two times, and 7.3% in settlements that were not reached. ^¶^ The amount of uninhabited area increased during August 2016–May 2019 because of population migration from insurgent-held areas to accessible areas.

## Routine Childhood Immunization

National coverage levels for the third dose of poliovirus vaccine (Pol3) delivered through routine immunization services by age 12 months have been <60% since 2002,* with lower rates in northern states. A 2016 survey indicated that Pol3 coverage nationally was 33% and <25% in seven of 13 northern states (*4*).

## Poliovirus Surveillance

**Acute flaccid paralysis surveillance.** The quality of polio surveillance is assessed by nonpolio AFP (NPAFP) rates and stool collection adequacy.^†^ Targets for Nigeria are an NPAFP rate of three or more cases per 100,000 population aged <15 years per year and stool collection adequacy ≥80% of AFP cases. In 2018, the national NPAFP rate was 9.6, and stool adequacy was 95%. As of May 31, 2019, the annualized national 2019 NPAFP rate was 8.0, and stool adequacy was 95%. In Borno, in 2018, the NPAFP rate was 24.5 with 85% stool adequacy; the annualized 2019 NPAFP rate is 19.6 and stool adequacy is 87%.

The destruction of health facilities after the insurgency disrupted health facility–based surveillance in Borno. Community informants help identify AFP cases in insurgent-held areas in Borno, particularly since February 2018. As of May 31, 2019, a total of 1,018 community informants in Borno reported 220 verified AFP cases during 2018–2019. Stool specimens are obtained when patients with AFP and their families temporarily leave insurgent-held areas for evaluation in safe areas; in 2018, stool adequacy for these AFP cases was 61% and in 2019, 79% to date.

**Environmental surveillance.** To supplement AFP surveillance, sewage samples are tested for polioviruses (*5*). During January 2018–May 2019, 25 (68%) of Nigeria’s 37 states had at least one environmental surveillance site for a total of 113 functional sites; no WPV and 85 cVDPV2 isolates were detected through environmental surveillance.

## cVDPV2 Outbreaks

Since 1988, widespread use of trivalent OPV (tOPV, containing Sabin serotypes 1, 2, and 3) reduced the number of polio cases >99% globally. WPV type 2 was declared globally eradicated in 2015 (*6*,*7*). In low-immunization settings, transmission of attenuated Sabin poliovirus contained in OPV can result in genetic reversion to VDPVs that can cause paralysis. When community transmission occurs, VDPVs are categorized as circulating (cVDPVs) (*8*). During 2006–2015, >94% of confirmed cVDPVs worldwide were cVDPV2 (*8*). To decrease the risk for cVDPV2, a globally synchronized switch from tOPV to bivalent OPV (bOPV, containing serotypes 1 and 3) occurred in April 2016 (*8*). Injectable inactivated poliovirus vaccine (IPV, containing serotypes 1, 2, and 3) was introduced into all OPV-using countries to provide individual protection from type 2 poliovirus paralysis (*9*). Both vaccines confer individual protection; however, IPV does not decrease fecal poliovirus shedding among children with infection, whereas OPV induces intestinal immunity and prevents shedding. Monovalent OPV type 2 (mOPV2) vaccine is available for cVDPV2 outbreak response (*10*). Since January 2018 (as of June 25, 2019), two independent cVDPV2 outbreaks have occurred (Table 1).

**TABLE 1:** Number of circulating vaccine-derived poliovirus type 2 (cVDPV2) cases, by acute flaccid paralysis (AFP) cases and environmental surveillance (ES) isolates in affected states — Nigeria, January 2018–May 2019*

Affected state	No. of cVDPV2 cases						cVDPV2 emergence outbreak source^†^	Date of most recent cVDPV2 case onset/ES specimen collection	
	Jan–Dec 2018		Jan–May 2018		Jan–May 2019				
	AFP	ES	AFP	ES	AFP	ES		AFP	ES
Bauchi	0	5	0	0	0	0	NIE-JIS-1	—^§^	Nov 5, 2018
Borno	6	5	0	0	1	16	NIE-JIS-1	Feb 14, 2019	Apr 2, 2019
Gombe	0	1	0	1	0	0	NIE-JIS-1	—	Apr 9, 2018
Jigawa	4	8	3	7	0	0	NIE-JIS-1	Oct 13, 2018	Jun 20, 2018
Kaduna	1	3	0	0	0	0	NIE-JIS-1	Sep 10, 2018	Dec 11, 2018
Kano	0	1	0	0	0	2	NIE-JIS-1	—	Mar 6, 2019
Katsina	16	0	0	0	0	0	NIE-JIS-1	Oct 22, 2018	—
Kwara	1	0	0	0	5	13	NIE-JIS-1	Mar 29, 2019	May 23, 2019
Lagos	0	1	0	0	0	6	NIE-JIS-1	—	May 10, 2019
Niger	0	0	0	0	1	0	NIE-SOS-3	Mar 18, 2019	—
Ogun	0	0	0	0	1	0	NIE-JIS-1	Mar 9, 2019	—
Sokoto	0	14	0	14	0	0	NIE-SOS-3	—	Jun 26, 2018
Taraba	1	0	0	0	0	0	NIE-JIS-1	Nov 2, 2018	—
Yobe	5	8	1	1	0	2	NIE-JIS-1	Nov 21, 2018	Feb 20, 2019
**Total**	**34**	**46**	**4**	**23**	**8**	**39**	**—**	**—**	**—**

**Abbreviation:** AFP = acute flaccid paralysis.

* As of June 25, 2019. In 2017, no WPV nor cVDPV2 cases or isolates were reported.

^†^ After the global switch from trivalent oral poliovirus vaccine (tOPV, containing Sabin types 1, 2, and 3) to bivalent OPV (bOPV, containing types 1 and 3), new emergences of cVDPV2 are identified by a three-letter country code, followed by three letters representing either state, province, or region, and a digit indicating the outbreak number in that state, province, or region.

^§^ Dashes indicate not applicable.

**Emergence in Jigawa State.** Eight cVDPV2-positive sewage samples collected during January 10–October 17, 2018, in Jigawa were genetically linked to four cVDPV2 cases with paralysis onset during April 15–October 13, 2018. This outbreak has spread to 11 other states, totaling 41 cVDPV2 cases and 71 sewage isolates. Genetically related poliovirus was also isolated from 11 AFP patients in Niger Republic, with onset July 18, 2018–April 3, 2109, and from one sewage sample collected April 4, 2019, in Cameroon.

**Emergence in Sokoto State**. A VDPV2 isolate was identified in a sewage sample collected January 30, 2018, in Sokoto; subsequent samples from three sites in 2018 yielded genetically related cVDPV2s. Genetically related cVDPV2 was isolated from an AFP patient in Niger State, with onset March 18, 2019.

## Vaccination Activities

In 2018, two national supplementary immunization activities (SIAs) with bOPV,^§^ one subnational SIA with bOPV in five states, two subnational SIAs in seven states using bOPV and fractional IPV, and three subnational SIAs in two states using bOPV and fractional IPV were conducted. One subnational SIA using bOPV has been conducted in seven states to date in 2019. Two subnational SIAs were conducted in two states using bOPV and fractional IPV. Gombe was the only state with three subnational SIAs to date in 2019 (Table 2).

**TABLE 2:** Number of supplementary immunization activities (SIAs) by state, vaccine formulation, and quality assessment of response SIAs — Nigeria, January 2018–May 2019*

State	2018			2019			Date of most recent mOPV2 SIA, 2019	% LGAs passing 90% threshold on LQAS^§,¶^
	bOPV	bOPV + fIPV^†^	mOPV2	bOPV	bOPV + fIPV	mOPV2		
Abia	2	—**	—	—	—	—	—	—
Adamawa	4	—	1	1	—	2	May 4	80–100
Akwa Ibom	2	—	—	—	—	—	—	—
Anambra	2	—	—	—	—	—	—	—
Bauchi	3	1	5	1	—	2	May 4	73–100
Bayelsa	2	—	—	—	—	—	—	—
Benue	2	—	1	—	—	1	Jan 26	89–100
Borno	4	1	2	1	—	1	May 25	87–100
Cross River	2	—	—	—	—	—	—	—
Delta	2	—	—	—	—	—	—	—
Ebonyi	2	—	—	—	—	—	—	—
Edo	2	—	—	—	—	—	—	—
Ekiti	2	—	—	—	—	1	May 18	100
Enugu	2	—	—	—	—	—	—	—
Federal Capital Territory	3	—	1	—	—	1	Jan 29	50–67
Gombe	3	—	3	1	2	2	Apr 27	73–100
Imo	2	—	—	—	—	—	—	—
Jigawa	3	1	4	—	2	1	Apr 27	44–85
Kaduna	3	—	1	—	—	2	Apr 13	80–90
Kano	3	1	3	—	—	3	May 25	78–100
Katsina	3	1	3	1	—	2	May 4	40–90
Kebbi	2	—	1	—	—	2	April 13	73–93
Kogi	2	—	—	—	—	—	—	—
Kwara	2	—	—	—	—	4	May 25	20–60
Lagos	2	—	—	—	—	1	May 18	38
Nasarawa	3	—	1	—	—	1	Jan 29	20–70
Niger	2	—	1	—	—	3	May 18	70–90
Ogun	2	—	—	—	—	1	May 18	50
Ondo	2	—	—	—	—	1	May 18	88
Osun	2	—	—	—	—	1	May 18	100
Oyo	2	—	—	—	—	3	May 18	50–100
Plateau	2	—	1	—	—	2	May 4	78–100
Rivers	2	—	—	—	—	—	—	—
Sokoto	3	1	5	—	2	2	Apr 13	75–100
Taraba	3	—	1	1	—	2	May 4	94–100
Yobe	4	1	3	1	1	1	May 25	71–88
Zamfara	4	—	1	—	—	2	Apr 13	40–60

**Abbreviations:** bOPV = bivalent oral poliovirus vaccine; fIPV = fractional dose inactivated poliovirus vaccine; LGAs = local government areas; LQAS = lot quality assurance sampling; mOPV2 = monovalent oral poliovirus vaccine type 2.

* As of June 25, 2019.

^†^ bOPV contains types 1 and 3; fIPV is an intradermal administration of 0.10 ml of IPV.

^§^ LQAS is a random sampling methodology used to assess quality of vaccination campaigns.

^¶^ Among all sampled LGAs at state level for all mOPV2 outbreak response SIAs.

** Dashes indicate not applicable.

Since December 2016, little change has occurred in the areas not accessible by standard house-to-house SIA teams in Borno. Two novel approaches for immunizing children in insurgent-held areas in Borno were implemented (*2*). Reaching Every Settlement utilizes security escorts to enable vaccinators to reach children in some settlements in insurgent-held areas, and Reaching Inaccessible Children enables vaccination of children by trained military personnel in settlements only accessible by these personnel (*2*). Satellite imagery is used to estimate population sizes in settlements and vaccination team movements are tracked using geographic information systems, providing data on geographic reach by these immunization approaches (Figure). Since January 2018, approximately 140,000 children in insurgent-held settlements were vaccinated during 13 Reaching Every Settlement rounds with bOPV and approximately 85,000 children aged <5 years were vaccinated with mOPV2 in response to cVDPV2 outbreaks. During five Reaching Inaccessible Children rounds, 71,370 children were vaccinated with bOPV. As of May 2018, among approximately 104,330 children aged <5 years remaining in insurgent-held areas, 43,840 (42.0%) have received at least 1 bOPV dose. Most unreached children reside in settlements scattered over a wide geographic area. Overall, among children aged <5 years in insurgent-held settlements that have been reached, 79.8% and 26.2% have been offered ≥3 doses of bOPV by Reaching Every Settlement or Reaching Inaccessible Children rounds, respectively.

During January 2018–May 2019, Nigeria conducted multiple mOPV2 outbreak response SIAs in states affected by cVDPV2 and neighboring states (Table 2). The quality of outbreak response SIAs as assessed by post-campaign lot quality assurance sampling surveys has been variable; the national average for mOPV2 SIAs ranged from 64% to 90% of sampled local government areas reaching the target 90% threshold of estimated proportion of children vaccinated. A limited number of mOPV2 doses have been given in insurgent-held areas by Reaching Every Settlement during mid-2016–mid-2019; therefore, approximately 104,330 children aged <5 years have had no exposure to type 2 OPV.

## Discussion

During 2003–2014, Nigeria reported the majority of WPV cases in Africa and was the origin of most WPV importation outbreaks (*1*,*6*). Polio eradication activities were aggressively enhanced in 2012–2014. The last WPV type 3 isolated worldwide was in Nigeria in November 2012. The patient with the most recent WPV1 case in Nigeria had paralysis onset in August 2016 (*2*), even as previously silent areas in Borno have incrementally increased surveillance with community informants. Progress in decreasing the number of unvaccinated children in insurgent-held areas resulted from improved vaccination reach by novel approaches and net population migration from insurgent-held areas to accessible areas. Additional surveillance sensitivity assessments in Nigeria and other African countries are underway, potentially to allow certification of interruption of WPV transmission by the African Regional Certification Commission in 2020. However, active cVDPV2 transmission continuing into 2020 in Nigeria or eight other countries on the continent with active cVDPV2 outbreaks might complicate the certification process.

Nigeria experienced multiple cVDPV2 outbreaks during 2005–2015 as well as ongoing transmission after cVDPV2 importation in 2013 because of vulnerability to emergence and spread of cVDPV2 from the predominant use of mOPV1, mOPV3 and bOPV during 2005–2014 SIAs, coupled with chronically low routine tOPV coverage (*6*). In addition, tOPV SIAs before the tOPV-to-bOPV switch were not sufficiently effective in all areas.

Children remaining in insurgent-held areas of Borno have remained inaccessible to mOPV2 administration by standard house-to-house SIAs for the cVDPV2 outbreak response; administration of mOPV2 in those areas will require Reaching Every Settlement and Reaching Inaccessible Children. Although mOPV2 is the tool to stop cVDPV2 outbreaks, it also carries the risk of seeding new emergences of cVDPV2 in areas with low-quality SIAs. Increased efforts for appropriate planning and supervision of subsequent SIAs will be important in ensuring optimal response quality necessary to interrupt cVDPV2 transmission and emergence.

**Summary**

**What is already known about this topic?**

The latest wild poliovirus (WPV) case in Nigeria occurred in August 2016 and was reported in September 2016, in Borno State.

**What is added by this report?**

The number of children living in insurgent-held areas in Borno who have not had access to poliovirus vaccines was reduced by 87% during December 2016–May 2017. Trained community members living in insurgent-held areas have reported suspected polio cases with no WPV identified on virologic testing, which suggests that WPV transmission might have been interrupted in Nigeria. However, outbreaks caused by type 2 circulating vaccine-derived poliovirus (cVDPV2) are spreading internationally.

**What are the implications for public health practice?**

Improved polio mass campaign quality is required to achieve interruption of all cVDPV2 transmission in Nigeria.

## Footnotes

* https://data.unicef.org/topic/child-health/immunization/.

† An annual non-polio AFP detection rate of ≥1 case per 100,000 population aged <15 years in countries in WHO regions certified as polio-free, or ≥2 in all other countries reflects the sensitivity of AFP surveillance. Stool adequacy refers to the collection of adequate stool specimens (i.e., two stool specimens collected >24 hours apart, within 14 days of paralysis onset, with arrival at the laboratory in good condition [cool and without leakage or desiccation]) from ≥80% of reported AFP patients and reflects the quality of case investigation. http://polioeradication.org/polio-today/polio-now/surveillance-indicators/.

§ SIAs are mass immunization campaigns conducted over several days to boost population immunity in areas with weak routine childhood immunization services and suboptimal coverage. The goal of SIAs is to reach every child aged <5 years with OPV, regardless of their vaccination status. http://polioeradication.org/who-we-are/strategic-plan-2013-2018/supplementary-immunization/.
